# Reaction injection molding of hydrophilic-in-hydrophobic femtolitre-well arrays

**DOI:** 10.1038/s41378-019-0065-2

**Published:** 2019-06-03

**Authors:** Reza Zandi Shafagh, Deborah Decrop, Karen Ven, Arno Vanderbeke, Robert Hanusa, Jolien Breukers, Gaspard Pardon, Tommy Haraldsson, Jeroen Lammertyn, Wouter van der Wijngaart

**Affiliations:** 1 Department of Micro- and Nanosystems, KTH Royal Institute of Technology, Stockholm, Sweden; 2 Department of Biosystems, KU Leuven, Willem de Croylaan 42, 3001 Leuven, Belgium

**Keywords:** Nanoscale devices, Nanoscale materials

## Abstract

Patterning of micro- and nanoscale topologies and surface properties of polymer devices is of particular importance for a broad range of life science applications, including cell-adhesion assays and highly sensitive bioassays. The manufacturing of such devices necessitates cumbersome multiple-step fabrication procedures and results in surface properties which degrade over time. This critically hinders their wide-spread dissemination. Here, we simultaneously mold and surface energy pattern microstructures in off-stoichiometric thiol-ene by area-selective monomer self-assembly in a rapid micro-reaction injection molding cycle. We replicated arrays of 1,843,650 hydrophilic-in-hydrophobic femtolitre-wells with long-term stable surface properties and magnetically trapped beads with 75% and 87.2% efficiency in single- and multiple-seeding events, respectively. These results form the basis for ultrasensitive digital biosensors, specifically, and for the fabrication of medical devices and life science research tools, generally.

## Simultaneous microstructuring and surface modification of microfluidic bioreactors

Researchers from Sweden and Belgium have developed a simplified technique to create microfluidic devices with patterns of modified surface properties. Microfluidic devices, such as the one demonstrated here by a team led by Wouter van der Wijngaart, from KTH Royal Institute of Technology, Sweden, utilize polymer microstructures with hydrophobic and hydrophilic properties to manipulate fluids on a tiny scale. This enables ‘lab-on-a-chip’ experiments with a diverse array of applications. However, current devices suffer from complicated fabrication processes and a limited lifespan. van der Wijngaart and his team used the polymer ‘off-stoichiometric thiol-ene’ to merge a rapid replication technique called reaction injection molding with a method to self-assemble hydrophobic and hydrophilic micropatterns in a single, rapid fabrication step. The researchers hope that their results will form the basis for future microfluidic chip manufacturing and lead to the creation of ultrasensitive life science research tools.

## Introduction

Microstructured surfaces and microfluidic components are increasingly important for a diverse range of biological and clinical applications, e.g., in protein and cell studies^1–3^. Using polymers for such applications is often preferred over glass or silicon because of the soft material properties and the ease of device replication. Whereas micro-injection molding (IM) and micro-reaction injection molding (RIM) enable industrial-scale microstructuring of low-cost polymer materials^4^, the lack of a simple and straightforward approach for surface modification remains a limiting factor^5^. Microscale hydrophilic and hydrophobic patterning is commonly achieved via back-end processing techniques, e.g., plasma treatment, grafting and chemical coating^6,7^, which are multi-step, cumbersome, and subject to degradation over time^8^. Furthermore, surface modification of 3D patterns and control over surface topography remain unresolved challenges.

Hydrophilic-in-hydrophobic (HIH) femtolitre-well arrays for digital bioassays are an example of structures that would greatly benefit from simpler fabrication techniques. Digital bioassays commonly exploit arrays of microwells as their hardware and can extensively benefit from selective surface energy patterning^9^. In such systems, the sample of interest is compartmentalized in well reactors amenable to high-throughput screening or online monitoring^10^. Such systems enable the ultra-sensitive detection of target molecules on a single-molecule level^11^. A common solution for efficient isolation of wells is to provide hydrophilic wells in a hydrophobic field. In bead-based digital assays, each well acts as a microchamber that traps a single bead functionalized with bioreceptors and surrounded by a micro-droplet of reagent. Femtolitre-well arrays for bead seeding in digital bioassays were previously fabricated mainly using cleanroom-based techniques^9,12–14^, poly(dimethylsiloxane) (PDMS) stamp imprinting^15^, or IM^16,17^. However, these materials and fabrication methods are expensive, not scalable, or do not result in defined surface energy features, the latter resulting in an inefficient bead seeding, leading to limited assay performance^18^.

Thiol-ene alternating copolymer constitutes an important class of thermosetting materials with a broad range of applications in optics, electronics, and biomedicine^19,20^. The UV polymerization and click reaction in these polymer networks enable rapid, efficient and by-product free reaction with often no need of solvent use. Also, the delayed gelation in thiol-ene polymerization leads to low shrinkage and low residual stress and enhances replication precision during molding^21^. Off-stoichiometric thiol-ene (OSTE) imparts additional features such as natively reactive surfaces, which offers a diverse spectrum of applications including adhesive-free bonding^22,23^ or direct binding of biomolecules^24^. Hydrophilic and hydrophobic moieties within an OSTE polymer precursor spontaneously self-assemble on areas of a master surface that have matching surface energies^25^. Sandström et al.^26^ reported micro-RIM of off-stoichiometric thiol-ene epoxy thermosetting polymers as a rapid processing technique that results in high-replication fidelity and low-residual stress.

Here, we demonstrate a molding technique capable of microstructuring and 3D in situ surface energy patterning in a single step. We use this platform for high-throughput, scalable replication of HIH femtolitre-well arrays and demonstrate a record-high seeding efficiency of magnetic beads in femtolitre-well arrays.

## Results and discussion

**Fig. 1:**
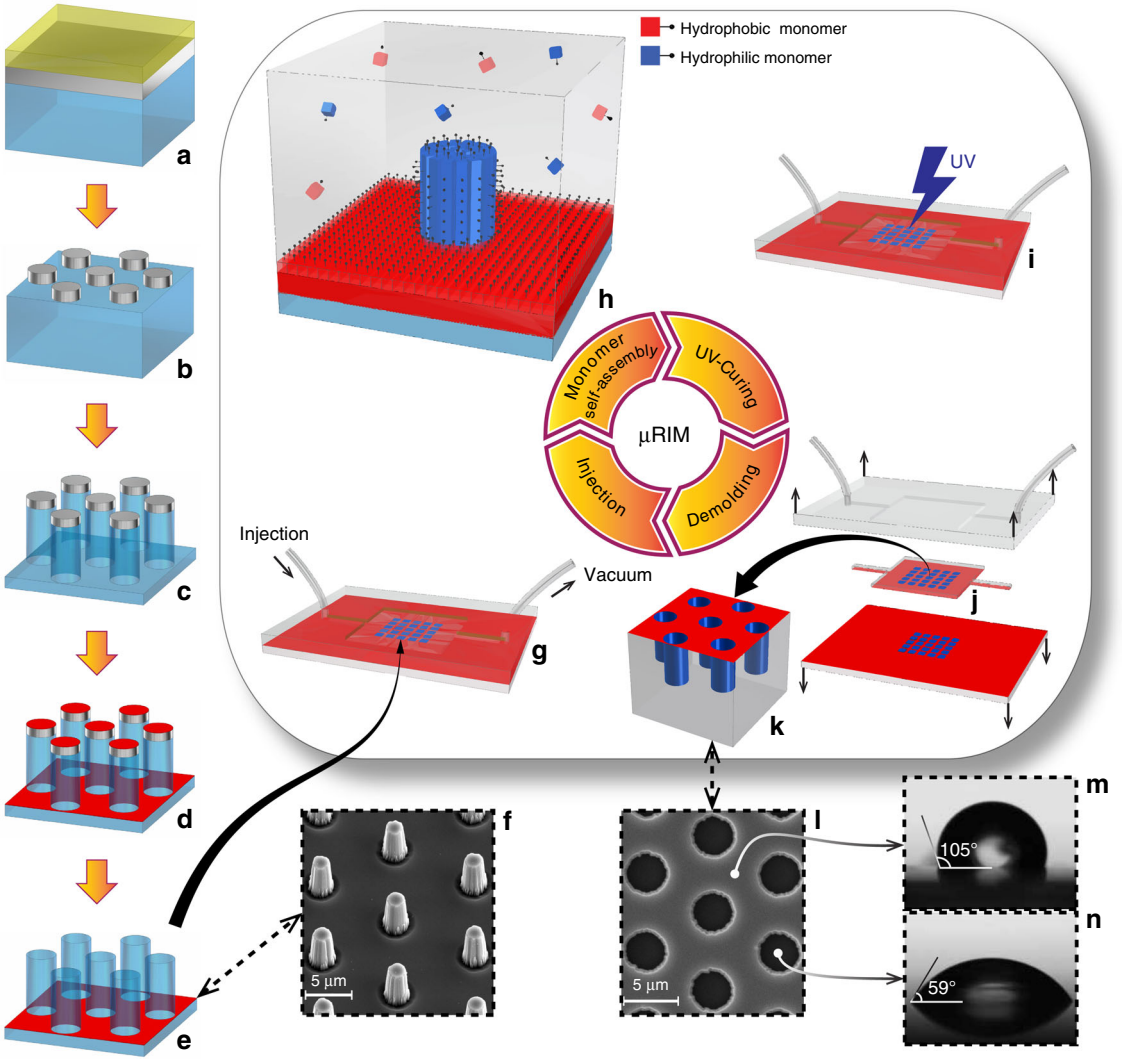
Schematic of the mold insert fabrication. **a**–**e** Schematic of the RIM replication using area-selective monomer self-assembly (**g**–**k**); and the resulting structures and surface energies (**f**, **l**–**n**). Blue color indicates hydrophilic surfaces; red color hydrophobic surfaces. **a** Fused silica substrate (blue) coated with Cr (gray) and photoresist (yellow). **b** Patterned Cr after photolithography and wet etching. **c** Fused silica pillars with Cr on top after dry etching. **d** Fused silica pillars with Cr and Teflon on top after Teflon (red) coating. **e** Finalized mold with hydrophilic pillar arrays in hydrophobic background after Cr lift-off. **f** Perspective SEM image of a mold insert with 3.5 μm diameter micropillars. **g** Mold assembly and precursor injection. **h** The area-selective self-assembly of monomers on mold surfaces with corresponding surface energy. **i** UV-curing. **j** Replica demolding. **k** Detail of HIH wells in the replica. **l** Top view SEM image of replicated HIH wells with 3.5 μm diameter and 8.5 μm pitch. **m**, **n** Contact angle measurements on corresponding hydrophobic and hydrophilic surfaces of non-structured polymer parts after 60 min incubation time

Figure 1 illustrates the specifically developed mold fabrication process, the replication process that combines RIM of OSTE with area-selective monomer self-assembly, and the resulting replicas and their surface energies. We developed a two-part HIH mold consisting of a milled Al half with good thermal conductivity, and a UV transparent microstructured half consisting of fused silica and Teflon^TM^. Using a hard mask is compatible with industrial injection molding settings in which a material of high Young-modulus circumvents the deformation and collapse of delicate microscale mold features. This is in contrast to use of PDMS, a prevalent material of choice in academia, which is prone to deformation and subsequent feature distortion during the molding process and therefore not easily applicable to commercial set-ups.

The fabrication method for the silica/Teflon^TM^ mold half requires only one photolithography step, resulting in a self-aligned structural and surface energy micropattern where exposed silica provides hydrophilic surfaces and Teflon^TM^ hydrophobic surfaces (Fig. 1a–e). The molds contain a 5 × 5 matrix of micropillar arrays, with a total of 1,843,650 circular pillars, with diameters in the range of 2.5–4.5 μm, and a centre-to-centre pitch in the range of 7–9 μm (Fig. 1f, g; see Fig. S1 for mold design details). The micropillar heights were in the range 3–4.5 μm. We injected an OSTE-based precursor, containing hydrophobic and hydrophilic chemical moieties, into the mold. During incubation in contact with the mold, the chemical moieties in the polymer network self-assemble on mold surface sections with matching surface energy. Cross-linking of the precursor during a 15 s UV exposure fixates the replica microstructure and its surface energy patterns, after which the replica is demolded. A mold consisting of micropillar arrays thus enables in situ surface energy patterned HIH femtolitre-well arrays with hydrophilic well bottom and sidewalls and hydrophobic interspacing. We observed a high-replication fidelity regarding geometry, size, and defectivity of the microwell arrays during microscopy inspection and optical profiling (Fig. S3). Contact angle measurements on larger planar features substantiate the successful mimicking of surface energy from the molds to the counterpart replica surfaces. The contact angle (CA) of the hydrophobic replica surfaces increases with the incubation time between injection and UV curing, from 90° ± 2.8 for no incubation to 104° ± 2.3 when incubating for 60 min (Fig. S2). The CA of hydrophilic replica surfaces was 59° ± 2.6, independent of the incubation time. The surface energy of the fully cured replicas was re-examined after 11 months storage of the samples in our ambient laboratory conditions (Table S1).

The unique benefit of our fabrication approach is the simultaneous molding and selective and stable surface energy patterning of microstructures in a single process step, while inheriting all benefits from RIM processing, including short-cycle time, facile processing, low tooling cost of RIM settings along with a surface reactive polymer for additional covalent bonding when fluidic integration is intended. The rapid cycle time, limited mainly by the 15 s UV exposure, constitutes by far the most rapid HIH production method reported. We speculate that the cycle time can be reduced to be on par with the typical standard injection molding cycle times, i.e., 2–5 s, by using a more rigid mold material such as quartz, by a vertical demolding set-up, or by adding more photoinitiator in the polymer precursor.

The dynamic range of a digital bioassay is related to the number of microwells, with the demonstrated array size of 10^5^–10^7^ wells theoretically enabling digital target detection with a dynamic range of 6–8 orders of magnitude^27^. The demonstrated well size and pitch in the µm range are small enough to facilitate integration of large arrays, but large enough for optical detection of individual wells. A mold pillar height/depth aspect ratio of 1/1 results in low-defectivity in terms of pillar collapse while providing a sufficient depth of the wells for trapping beads. For mold pillar aspect ratios >1, the manual and nonvertical demolding led to their occasional collapse.

The CA measurements demonstrate the surface energy pattern replication. The reduced concentration of hydrophilic or hydrophobic moieties resulted in microstructured replicas with excellent functionality, but also in a dependence of the hydrophobic mimicking phenomenon on the incubation time. Nevertheless, even at zero incubation time, the hydrophobic surfaces feature an equilibrium CA equal to 90°, which proved sufficient for bead seeding and well sealing. Although the degree of hydrophilicity associated to the replica is lower than that of glass, it is sufficient for droplet entrapment inside the microwells. The CA on the hydrophobic polymer surface did not change significantly during long-term storage; that of the hydrophilic surface reduced from 59.3 ± 2.6° to 48.1 ± 2.2°. We attribute this increase of hydrophilicity to oxidation of the thiol groups when exposed to the ambient air^28^. Compared to postprocess surface modification techniques such as plasma treatment of PDMS, which is prone to fast recovery of surface energy, our method offers a far more stable surface energy definition with some degree of enhancement regarding the hydrophilic surfaces over time.

**Fig. 2:**
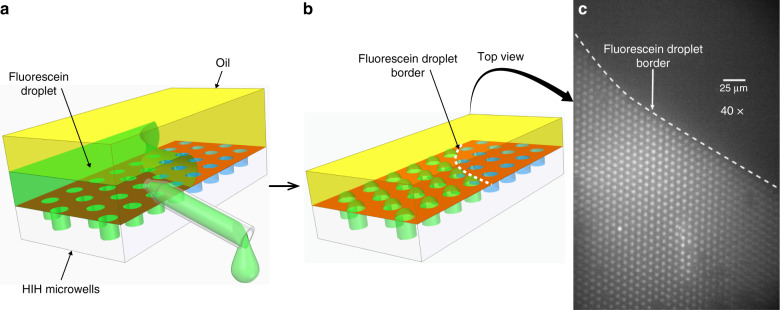
Isolation of femtolitre volume fluorescein microdroplets in microwell arrays. **a** Schematic of fluorescein and oil stacked on top of HIH microwells followed by the fluorescein removal. **b** Schematic of the resulting femtolitre fluorescein microdroplets trapped inside the wells. **c** Top view fluorescence microscopy image of fluorescein microdroplets inside HIH femtolitre-wells: the dark region in the top half of the image has been only in contact with oil whereas the lower half of the image is printed by the receding fluorescent droplet. The dashed line indicates the border of the fluorescein droplet shown in (**a**)

To demonstrate the hydrophilic nature of the bottoms of the microwells, we isolated fL-sized droplets of an aqueous solution of fluorescein in the microwells and sealed them with oil to prevent evaporation. Visualization of the aqueous fluorescein droplets and the absence of fluorescence at the interspacing surface confirms the HIH nature of the array (Fig. 2).

**Fig. 3:**
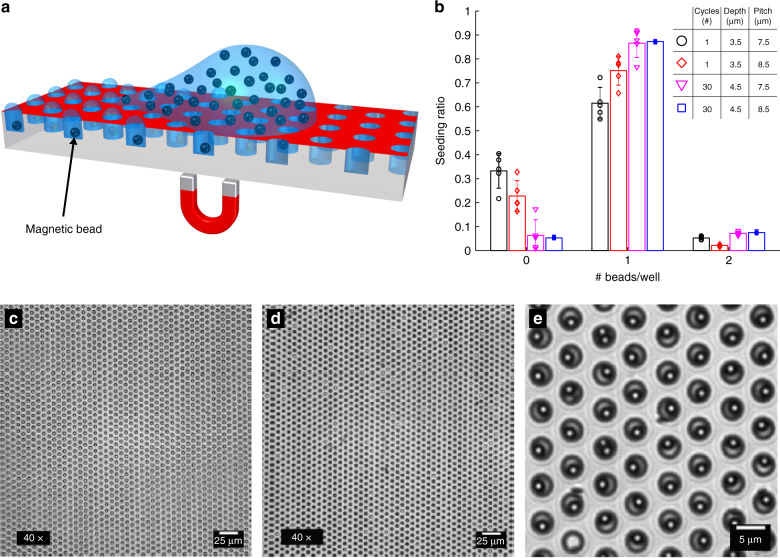
Magnetic bead seeding. **a** Schematic of the magnetic bead seeding on HIH microwell arrays. **b** The bead distribution in arrays with well diameter 3.5 μm and with varying array pitch and well depth, for single- and multiple-seeding cycles (*n* = 5). Dots indicate results from each experiment; columns indicate the average ratio of wells that contain a given number of beads; error bars mean ± s.d. **c**, **d** ×40 magnification bright field microscopy image of a microwell array before and after magnetic bead seeding, respectively. Bright spots indicate empty microwells and dark spots indicate filled microwells. **e** ×100 magnification bright field microscopy image of a microwell array after seeding, where one well is empty (bright), two wells contain two beads, and all other wells contain one bead

To investigate the potential of surface energy patterned microwells for digital bead-based bioassays, we seeded well arrays with magnetic beads (Fig. 3). All CA combinations were sufficient for bead seeding in surface energy patterned microwells, allowing minimizing the RIM incubation time. Digital counting of the magnetic beads revealed seeding efficiencies as high as 75.1 ± 6.0% for single-step seeding and 87.2 ± 0.3% for multiple-step seeding (Fig. 3b, Table S2).

The high-seeding efficiency reported here is ascribed to the successful surface energy patterning of the arrays and is of significance for bead-based bioassays, which require rapid and efficient bead seeding. Our results compare well with the estimated 40–50% gravity-based bead loading efficiency achieved in the SIMOA process^16^ and the maximum 35% efficiency obtained in previously reported single-step seeding^12^.

The dimensions of the demonstrated microwells are tailored to host a single magnetic bead with 2.7 μm diameter. Not to undermine the true digital nature of the assay, wells containing more than one bead must be excluded from the signal count during the image analysis, and their number should therefore remain limited. For well arrays with 8.5 μm pitch, we estimate trapping two beads in as little as 2.1 ± 0.4% of the wells after single-step seeding, and in 7.1 ± 0.8% after multiple-step seeding (Fig. 3e).

The RIM platform demonstrated here is adapted for use in laboratory settings, allowing a potential widespread dissemination of the technology. Because life science research typically relies on several tens of identical devices to verify the reproducibility of experiments, the rapid device replication offers an attractive alternative to the manual and error-prone per-part assembly of soft lithography-based devices.

Future work should examine the potential of RIM-based microwell arrays for digital bioassays aiming for single molecule detection. Furthermore, translating the current technology to industrial RIM standards needs a re-adjustment of the mold material, mold cleaning protocols, injection settings, and demolding.

## Conclusions

In conclusion, this work introduces reaction injection molding as a scalable platform suitable for use both in high-throughput and cost-effective manufacturing and in laboratory settings. We demonstrate the technique for the replication of microwell arrays. The distinct feature of simultaneous molding and surface energy patterning results in the structuring of hydrophilic-in-hydrophobic femtolitre well arrays capable of magnetic bead seeding with efficiencies of 75.1% and 87.2% for single-cycle and multiple-cycle seeding, respectively. This technology can pave the road towards successful commercialization of digital-based bioassays, specifically, and is amenable to diverse adaptations in medical devices and life science tools, where the spatial control of surface topology and properties is critical.

## Methods

### Mold fabrication

The RIM setup is comprised of two mold halves which come together to form the negative shape of the final chip. The first mold half is a 5 mm thick Al sheet (4.5 × 7.4 cm^2^) CNC milled (MiniMill GX, Minitech Machinery Corp., USA) with a 3.5 × 3.5 cm^2^ indentation to form a cavity. Inlet and outlet channels connect the cavity to the edges of the mold.

The second mold half consists of the mold insert, i.e., a 0.5 mm thick fused silica mold, also sized 4.5 × 7.4 cm², strengthened with a 2 mm thick piece of borosilicate glass glued to its backside. The fused silica structuring is illustrated in Fig. 1a–e. We started from 0.5 mm thick 4″ fused silica wafers and performed the subsequent steps. We evaporated 250 nm of Cr. We deposited the adhesion promotor Hexamethyldisilazane from the gas phase, spin-coated positive tone photoresist (AZ4533, AZ Electronic materials, Germany), and performed photolithography (Mask aligner, Karl Suss, Germany) with 6 s of UV exposure. We developed the resist in CD-26 Developer (Dow, Rohm-Haas supplier, Germany) for 85 s, followed by hard baking at 115 °C for 120 s, and O_2_ plasma ashing for 4 min at 150 W. We etched the Cr in a Cr etchant consisting of acetic acid: 3.92% w/w, water: 74.73% w/w, and diammonium cerium(IV) nitrate: 21.35% w/w. We stripped the photoresist and dry etched the wafer with DRIE (STS ICP Multiplex Advanced Oxide Etch) using H_2_, He and C_4_F_8_. We silanized and then teflonized the wafers, in which the silanization acts as an adhesion promoter for the subsequent Teflon^TM^ coating on the fused silica. We prepared a hydrophobic fluoroalkylsilane solution consisting of silane (Dynasylan F8263, Evonik Resource Efficiency GmbH, Essen, Germany): 2% w/w, ethanol: 93% w/w, and water: 5% w/w and spin coated it on the substrate at 3000 rpm for 60 s. We baked the substrates at 110 °C for 12 min. We diluted Teflon AF 1601 (Chemours, USA) with fluorinert FC-40 (Sigma-Aldrich, Germany) with the ratio of 1:6, respectively. We spincoated the Teflon solution on the substrates at 2000 rpm for 60 s, followed by baking at 180 °C for 10 min. A lift-off process was subsequently performed in Cr etchant solution to remove Cr and Teflon from the pillar tops.

The two mold halves are held together using an Al molding fixture that is 9 mm thick at its base, with sidewalls to align the mold halves. A pair of toggle clamps applies pressure to the mold from above. We attached silicone tubing with an ID of 1.5 mm and OD of 5 mm to the inlet and outlet channels of the mold. Two inlet and outlet ports, embedded into both sides of the Al mold body, enable injection and ventilation prior and during RIM experiment. We initially connected the outlet port to an in-house vacuum source (20 mbar) for degassing the mold cavity and avoiding air entrapment during RIM.

### OSTE precursor synthesis

We purchased the following monomers and initiator from Sigma-Aldrich, Germany: pentaerythritol tetrakis(3-mercaptopropionate) (PETMP), 1,3,5-triallyl-1,3,5-triazine-2,4,6(1H,3H,5H)-trione (TATATO), 3,3,4,4,5,5,6,6,7,7,8,8,9,9,10,10,10-heptadecafluorodecyl methacrylate (FDMA), 2-hydroxyethyl methacrylate (HEMA), and photo-initiator 1-hydroxycyclohexyl phenyl ketone (Irgacure 184). We synthesized the OSTE precursor with excess of allyl functional groups with the following subsequent steps: (i) mixing the allyl monomer TATATO with the photo-initiator (52.32 and 0.5 wt% of the final mixture, respectively), (ii) heating the mixture to 75 °C for 10 min to facilitate the dissolution of the solid photo-initiator, (iii) adding the thiol monomer PETMP (47.08 wt% of the final mixture), (iv) mixing and subsequent degassing, (v) adding HEMA and FDMA, both at 0.05 wt%, and (vi) mixing again.

### RIM process

The RIM process is illustrated in Fig. 1g–j. A 5 mL syringe was filled with OSTE precursor, equipped with a 1.20 mm by 40 mm Sterican blunt needle and actuated to purge air bubbles from the tip of the syringe and needle. The needle was fitted to the inlet tube, and the syringe was slowly actuated to inject the precursor into the cavity. To allow for shrinkage compensation during curing, we adjusted the volume of precursor injected to include the volume of the entire mold chamber and part of the outlet channel. We, thereafter, removed the syringe and needle. The hydrophilic and hydrophobic moieties in the precursor self-assembled on mold surfaces with matching surface energy, i.e., hydrophilic moieties self-assembled on pillars, resulting in hydrophilic replica surfaces in the wells, and hydrophobic moieties self-assembled on the Teflon substrate, resulting in a hydrophobic top surface of the replicas. We performed experiments in which we allowed incubation of the precursor in the setup for up to 60 min, while shielding from UV light, to allow the hydrophilic and hydrophobic moieties to diffuse to surfaces of the mold with matching surface energies. Thereafter, the precursor was cured for 15 s at 12.5 mW/cm^2^. After curing, we separated the mold halves using a custom demolding fixture consisting of four ejection pins and removed the replica from the mold using tweezers.

### CA measurements

We performed CA measurements using a Theta Lite optical tensiometer (TL100, Finland) on non-structured polymer parts replicated from flat, Teflon-coated and bare fused silica molds, respectively. We chose three random areas on each sample and calculated the average CA accordingly. We performed the CA measurements immediately after the RIM process and 11 months after the RIM process measurements to allow measuring surface energy recovery over time.

### 3D optical imaging

We used an optical profilometer (SENSOFAR S lynx with software SensoSCAN 6.4) for 3D optical cross-sectional imaging of micropillar and microwell arrays.

### Fluorescein isolation in the wells

We dragged a 30 μL droplet of a 0.25 mg/mL fluorescein solution (Sigma-Aldrich, Belgium) over the microwell array. We subsequently covered the droplet of fluorescein with a 180 μL droplet of PlusOne Drystrip Coverfluid oil (Sigma-Aldrich, Belgium) to prevent evaporation. Next, we removed the fluorescein from underneath the oil using a glass pipette tip, first dragging the fluorescein droplet away from the array, then pipetting the fluorescein out from underneath the oil. The sealed microwells were visualized using an inverted fluorescence microscope (Nikon TiEclipse, Japan), using a TITC filter (excitation 465–495 nm, emission 515–550 nm).

### Magnetic bead seeding

We purchased streptavidin-coated LodeStar 2.7 μm superparamagnetic beads with a stock concentration of 8 × 10^8^ beads/mL from Agilent Technologies (Santa Clara, CA, USA). To prepare the beads for seeding, 10 µL of the stock solution was washed three times in 150 μL of a mixture of phosphate-buffered saline (PBS), 0.1% bovine serum albumin, and 0.1% Tween-20. The beads were resuspended in 80 μL PBS containing 2% Tween-20. For multiple seeding events, we pipetted a 5 μL drop of this solution on the microwell array, and a plastic pipette tip was used to move the droplet back and forth across the array 30 times while a magnet was held underneath the array to attract the beads into the wells. After the 30 seeding events, we removed the magnet and pushed the droplet off of the array. In the case of a single seeding event, we moved the droplet across the array only once. We visualized the beads using an inverted fluorescence microscope in bright field mode (Nikon TiEclipse, Japan) and counted for five different samples.

Viewing with a ×40 objective allowed scanning a large area and distinguishing wells with and without beads, but did not allow distinguishing between wells with a single and those with multiple beads. At ×100 magnification it was feasible to distinguish between wells with zero, a single or two beads.

## Supplementary information

Supplementary Figure S1

Supplementary Figure S2

Supplementary Figure S3

Supplementary Figure S4

Supplementary Table S1 and Table S2
