# Supplementary figures and images for: Reaction injection molding of hydrophilic-in-hydrophobic femtolitre-well arrays
Source: Microsyst Nanoeng. 2019 Jun 3;5:25. doi: 10.1038/s41378-019-0065-2 (PMC6545322; doi:10.1038/s41378-019-0065-2)

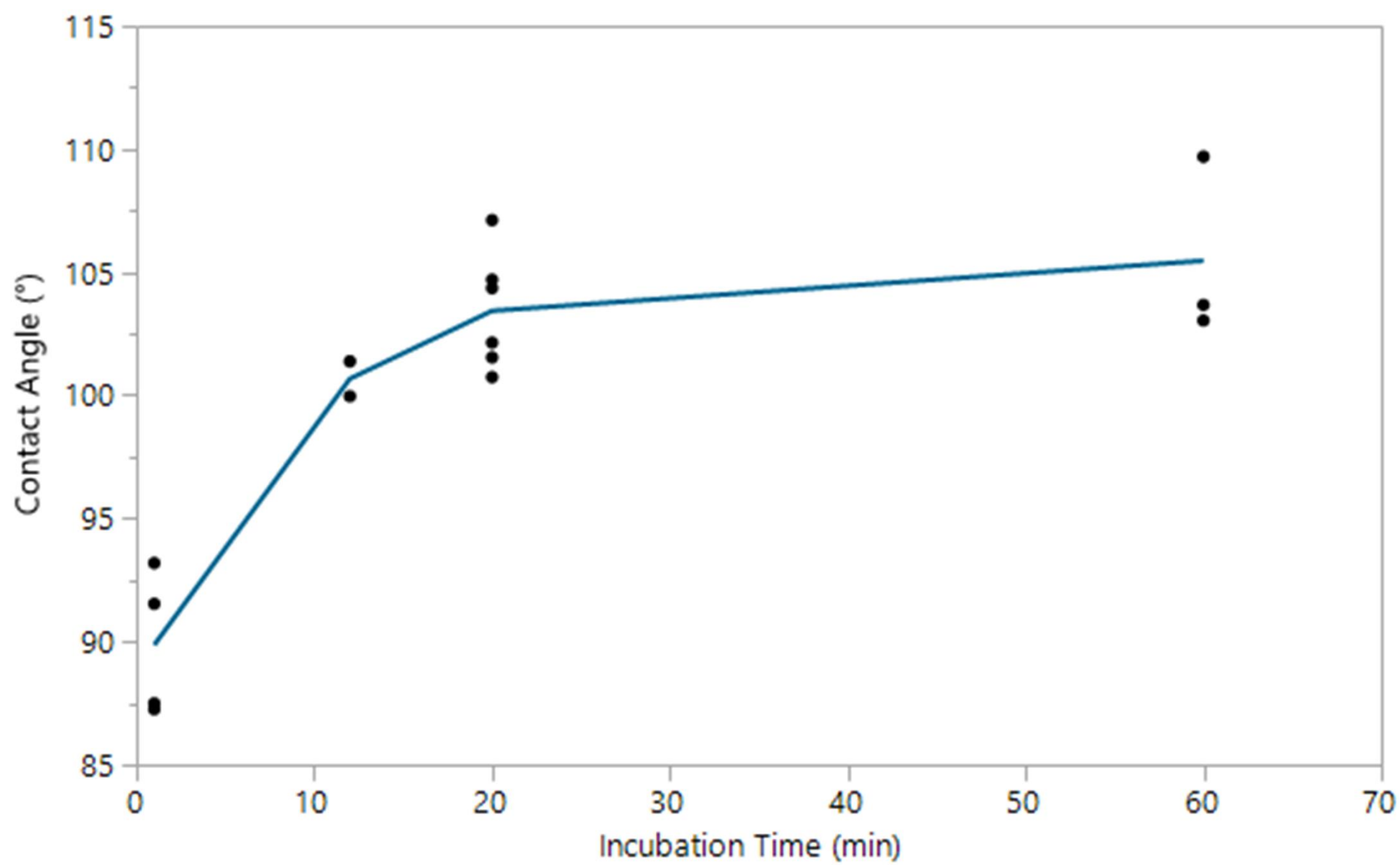

Supplement: Supplementary file 1 — Supplementary Figure S1 [file 41378_2019_65_MOESM1_ESM.pdf]

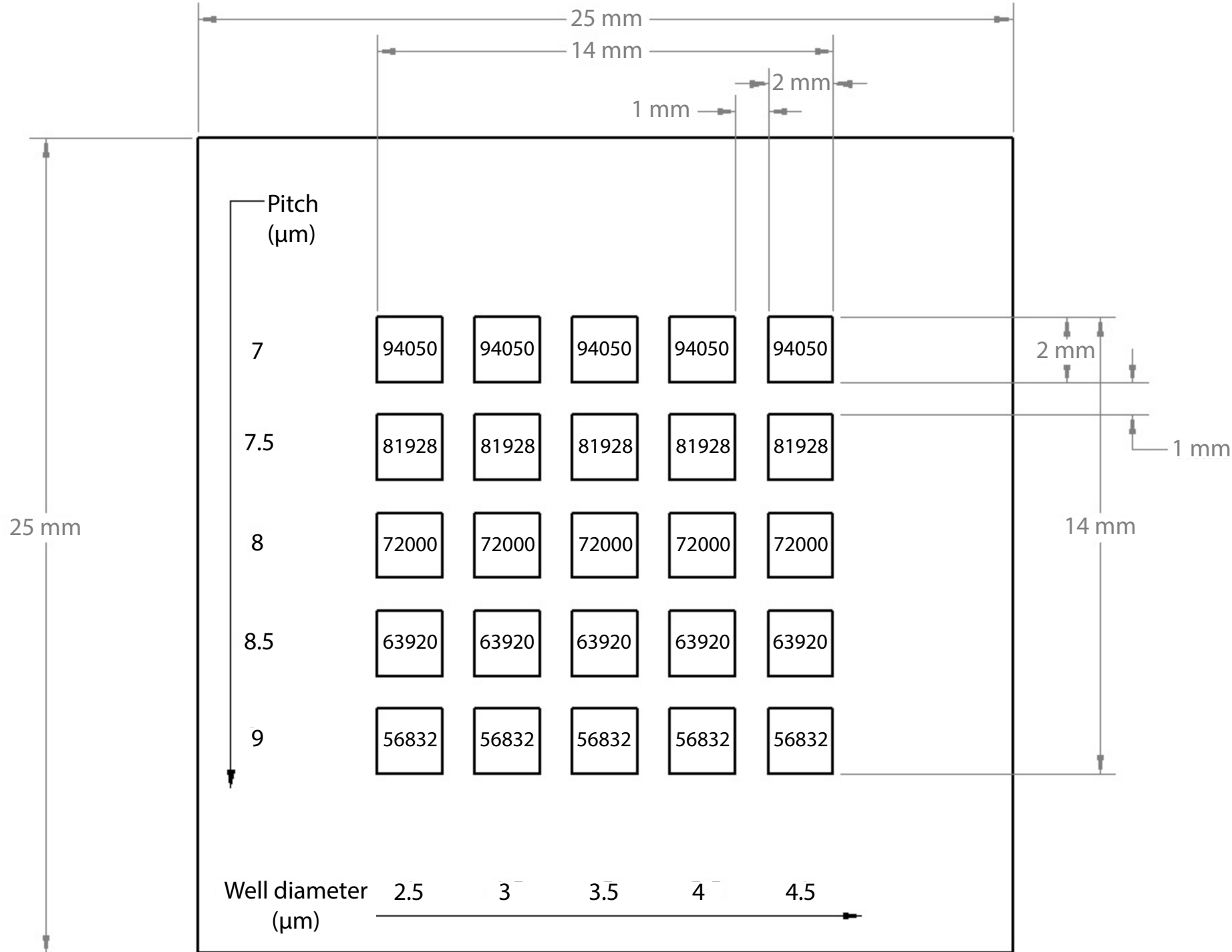

Supplement: Supplementary file 2 — Supplementary Figure S2 [file 41378_2019_65_MOESM2_ESM.pdf]

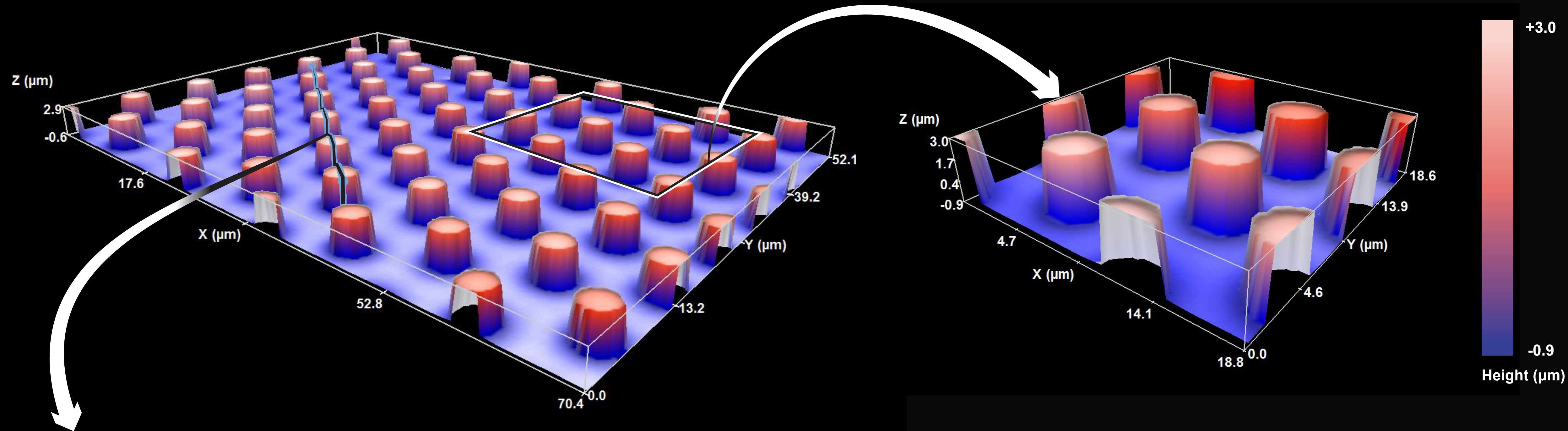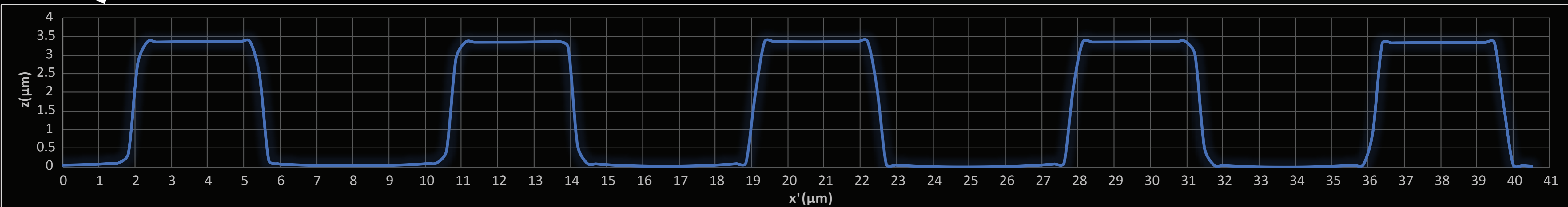

Supplement: Supplementary file 3 — Supplementary Figure S3 [file 41378_2019_65_MOESM3_ESM.pdf]

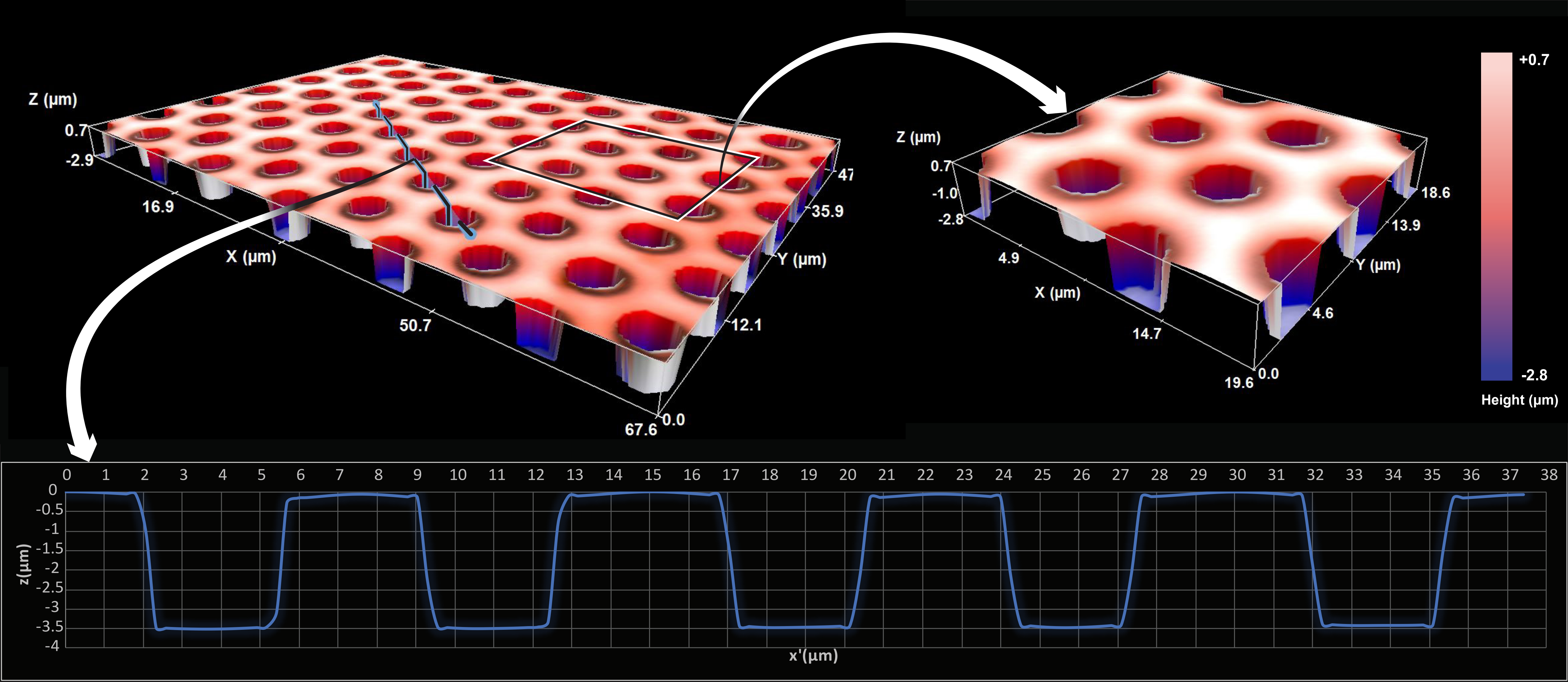

Supplement: Supplementary file 4 — Supplementary Figure S4 [file 41378_2019_65_MOESM4_ESM.pdf]
