# Supplementary material for: Reaction injection molding of hydrophilic-in-hydrophobic femtolitre-well arrays
Source: Microsyst Nanoeng. 2019 Jun 3;5:25. doi: 10.1038/s41378-019-0065-2 (PMC6545322; doi:10.1038/s41378-019-0065-2)
Supplement: Supplementary file 5 — Supplementary Table S1 and Table S2 [file 41378_2019_65_MOESM5_ESM.pdf]

Table S1: Contact angle measurements on replicas demolded from both Teflon and Glass molds, carried out immediately after demolding and 11 months later.

Measurement time	Immediately after RIM		11 months after RIM	
Mold material	Teflon	Glass	Teflon	Glass
Contact angle	103.4°	62.2°	105.6°	46.7°
	102.4°	57.1°	108.3°	47.1°
	106.8°	58.7°	103.8°	50.6°
Mean	104.2°	59.3°	105.9°	48.1°
SD	2.3°	2.6°	2.3°	2.2°

Bead count after magnetic bead seeding with a single seeding cycle on 5 arrays of both of 7.5 and of 8.5 µm pitch microwells with 3.5 µm diameter and 3.5 µm depth (top), and; after magnetic seeding with 30 seeding cycles on 5 arrays of both of 7.5 and of 8.5 µm pitch microwells with 3.5 µm diameter and 4.5 µm depth (bottom). The 40× magnification columns indicate the total number of wells counted and the number of those wells that were filled with at least one bead, as observed through a 40× magnification objective. The 100× magnification columns indicate the number of filled wells with 0 beads, 1 bead and 2 beads, as observed through a 100× magnification objective.

Bead count after magnetic bead seeding with a single seeding cycle on 5 arrays of both of 7.5 and of 8.5 µm pitch microwells with 3.5 µm diameter and 3.5 µm depth (top), and; after magnetic seeding with 30 seeding cycles on 5 arrays of both of 7.5 and of 8.5 µm pitch microwells with 3.5 µm diameter and 4.5 µm depth (bottom). The 40× magnification columns indicate the total number of wells counted and the number of those wells that were filled with at least one bead, as observed through a 40× magnification objective. The 100× magnification columns indicate the number of filled wells with 0 beads, 1 bead and 2 beads, as observed through a 100× magnification objective.

Table S2:
Bead count after magnetic bead seeding with a single seeding cycle on 5 arrays of both of 7.5 and of 8.5 µm pitch microwells with 3.5 µm diameter and 3.5 µm depth (top), and; after magnetic seeding with 30 seeding cycles on 5 arrays of both of 7.5 and of 8.5 µm pitch microwells with 3.5 µm diameter and 4.5 µm depth (bottom). The 40× magnification columns indicate the total number of wells counted and the number of those wells that were filled with at least one bead, as observed through a 40× magnification objective. The 100× magnification columns indicate the number of filled wells with 0 beads, 1 bead and 2 beads, as observed through a 100× magnification objective.

	40× magnification			100× magnification			
Pillar pitch	0 beads	$\geq$ 1 bead	total	0 beads	1 bead	2 beads	total
7.5 µm	850	1458	2308	75	305	30	410
	1020	1295	2315	82	304	26	412
	816	1477	2293	49	303	36	388
	515	1730	2245	51	292	24	367
	956	1349	2305	55	298	13	366
8.5 µm	405	1373	1778	7	269	13	289
	392	1389	1781	15	264	3	282
	675	1125	1800	9	278	1	288
	513	1282	1795	7	268	8	283
	328	1474	1802	15	263	13	291

	40× magnification			100× magnification			
Pillar pitch	0 beads	$\geq$ 1 bead	total	0 beads	1 bead	2 beads	total
7.5 µm	139	2269	2408	37	315	35	387
	117	2288	2405	33	320	14	367
	439	1973	2412	43	316	23	382
	16	2403	2419	26	337	14	377
	16	2394	2410	9	342	50	401
8.5 µm	117	1745	1862	9	267	15	291
	91	1778	1869	19	260	37	316
	96	1776	1872	15	264	24	303
	102	1764	1866	7	270	21	298
	105	1773	1878	12	267	18	297
